# Chest CT scan and alveolar procollagen III to predict lung fibroproliferation in acute respiratory distress syndrome

**DOI:** 10.1186/s13613-019-0516-9

**Published:** 2019-03-27

**Authors:** Annabelle Hamon, Ugo Scemama, Jérémy Bourenne, Florence Daviet, Benjamin Coiffard, Nicolas Persico, Mélanie Adda, Christophe Guervilly, Sami Hraiech, Kathia Chaumoitre, Antoine Roch, Laurent Papazian, Jean-Marie Forel

**Affiliations:** 1 Médecine Intensive Réanimation Détresses Respiratoires et Infection Sévères, AP-HM, CHU Nord, chemin des Bourrely, 13015 Marseille, France; 2 CEReSS - Centre for Studies and Research on Health Services and Quality of Life EA3279, Faculté de médecine, Aix-Marseille University, Boulevard Jean Moulin, Marseille, France; 3 Imagerie Médicale, AP-HM, CHU Nord, chemin des Bourrely, 13015 Marseille, France; 4 Médecine Intensive Réanimation des Urgences Médicales, AP-HM, CHU Timone, 13005 Marseille, France

**Keywords:** Acute respiratory distress syndrome, Fibroproliferation, CT scan, Scoring, Procollagen, Lung fibrosis

## Abstract

**Background:**

Lung fibroproliferation in ARDS patients is associated with mortality. Alveolar procollagen III (NT-PCP-III) is a validated biomarker of lung fibroproliferation. A chest CT scan could be useful for the diagnosis of lung fibroproliferation. The aim of this study was to identify lung fibroproliferative CT scan aspects in ARDS patients with high levels of NT-PCP-III.

**Results:**

This retrospective study included ARDS patients who had at least one assessment of alveolar NT-PCP-III and a chest CT scan within 3 days before or after NT-PCP-III determination. An alveolar level of NT-PCP-III > 9 µG/L indicated fibroproliferation. The CT scan was scored on interstitial and alveolar abnormalities. Each lobe was scored from 0 to 5 according to the severity of the abnormalities. The crude score and the corrected score (related to the number of scored lobes in cases of important lobar condensation or lobectomy) were used. One hundred ninety-two patients were included, for a total of 228 alveolar NT-PCP-III level and CT scan ‘couples’. Crude and corrected CT scan fibrosis scores were higher in the fibroproliferation group compared with the no fibroproliferation group (crude score: 12 [9–17] vs 14 [11–12], *p* = 0.002; corrected score: 2.8 [2.2–4.0] vs 3.4 [2.5–4.7], *p* < 0.001). CT scan fibrosis scores and NT-PCP-III levels were significantly but weakly correlated (crude score: *ρ* = 0.178, *p* = 0.007; corrected score: *ρ* = 0.184, *p* = 0.005).

**Conclusions:**

When the alveolar level of NT-PCP-III was used as a surrogate marker of histological lung fibroproliferation, the CT scan fibrosis score was significantly higher in patients with active lung fibroproliferation. Pulmonary condensation is the main limitation to diagnosing fibroproliferation during ARDS.

**Electronic supplementary material:**

The online version of this article (10.1186/s13613-019-0516-9) contains supplementary material, which is available to authorized users.

## Background

Acute respiratory distress syndrome (ARDS) is associated with a high mortality rate of 35% in intensive care units and 40% in hospitals [1]. Histological studies show that an early inflammatory phase is followed by a fibroproliferative repair phase, leading to either the resolution of ARDS or irreversible lung fibrosis [2]. It has been shown that this ARDS-associated lung fibrosis is linked to a poor outcome [3–5], an increase in the duration of mechanical ventilation [6] and an alteration in the quality of life [7]. Modulating excessive lung fibroproliferation could improve the ARDS prognosis. Corticosteroid treatments have been shown to be effective in improving the number of days alive without mechanical ventilation [8, 9], although the effect on mortality is still controversial [10–13]. All these points plead for the use of diagnostic tools for the early diagnosis of lung fibroproliferation during ARDS.

The gold standard for diagnosing ARDS-associated lung fibrosis remains an open lung biopsy (OLB) and histological examination, which is invasive and cannot be repeated easily [14]. Clinical parameters (oxygenation; compliance of respiratory system) remain insufficient and unspecific for the diagnosis. The N-terminal peptide of alveolar procollagen III (NT-PCP-III) is validated as an alternative diagnostic test [15]. A precursor of collagen III, NT-PCP-III represents the pulmonary turnover of collagen [16–18]. It has been shown that alveolar NT-PCP-III exceeding 9 µG/L on bronchoalveolar lavage (BAL) is associated with histological lung fibroproliferation [15].

Chest CT scan is less invasive that fibroscopic bronchoalveolar lavage and is available everywhere, conversely to NT-PCP-III. The radiologists describe frequently ground glass opacities, interlobular septal thickenings and sometime honeycombing in ARDS patients. The clinicians use frequently these findings for the lung fibrosis diagnostic in the clinical practice. In the context of idiopathic pulmonary fibrosis, a chest CT scan allows the diagnosis and quantification of fibroproliferative lung lesions [19, 20]. Several studies highlight its use in predicting morbidity and mortality during ARDS [6, 21, 22], although few studies have validated the diagnostic yield of CT scan for the diagnosis of fibroproliferation during ARDS. In the first 24 h of ARDS, Ichikado et al. [22] showed that a low lung fibroproliferation CT scan score was associated with a decrease in mortality (19.1 vs 57.9%, *p* < 0.0001) and an increase in the number of days alive and free of mechanical ventilation (14.3 vs 5.1 days, *p* < 0.0001). On the 14th day of ARDS evolution, Burnham et al. [23] showed the presence of CT scan signs of fibroproliferation in patients with the lowest static respiratory system compliance.

The main objective of the present study was therefore to quantify chest CT scan lesions in ARDS patients with a high alveolar level of NT-PCP-III, indicating a lung active fibroproliferation. The secondary aim was to determine the threshold for the CT scan fibrosis score compared to the alveolar level of NT-PCP-III.

## Methods

### Setting and participants

This study was designed retrospectively, but all the information needed to complete the datasheet was obtained prospectively. The study was carried out in the 14-bed intensive care unit of a teaching hospital from January 2013 to January 2017 and conducted according to French ethical law. The Institutional Review Board committee approved the research (Comité Informatique et Liberté AP-HM, 2017-0014). No interventional strategy was tested. Informed consent was not required due to the retrospective nature of the study. Patient information was anonymized before the analysis.

From a patient data registry, we selected all patients who, during the evolution of ARDS, had at least one dose of alveolar NT-PCP-III (obtained by performing a BAL) and a chest CT scan within a maximum interval of 3 days (i.e. BAL within 3 days before/after the chest CT scan). If a patient presented at least one of the following diagnoses, he/she was excluded: lung transplant, autoimmune diseases as vasculitis, chronic fibrotic lung disease or corticosteroid treatment (> 200 mg/day of hydrocortisone or equivalent at any moment during the month preceding inclusion). When one patient had multiple chest CT scan and alveolar NT-PCP-III determination ‘couples’, each case was included in the analysis.

### Measures

Bronchoalveolar lavage was performed according to a standardized procedure as part of clinical care. The BAL fluid was obtained from the most infiltrated lung area on chest X-ray according to a previously described technique [15]. Alveolar NT-PCP-III was measured using a radioimmunological method (UniQ-procollagen III Radioimmunoassay; Orion Diagnostica, Espoo, Finland). The alveolar NT-PCP-III threshold used to define lung fibroproliferation was > 9 µG/L [15]. The ‘lung fibroproliferation group’ was defined by an alveolar level of NT-PCP-III > 9 µG/L, and the ‘no lung fibroproliferation group’ was defined by an alveolar level of NT- PCP-III ≤ 9 µG/L.

Chest CT scans were realized using either a GE OPTIMA CT 660 system (GE Healthcare, Milwaukee, WI, USA) or a SIEMENS SOMATOM SENSATION 64 system (Siemens Medical Solutions, Forchheim, Germany). The CT scans were obtained in helical mode with inframillimetric section (kV: 100–120, depending on the patient’s body mass index; mAs: variable; pitch: 1.531/1; pixel spacing: 0.855 mm/0.855 mm; *Z* axis: 350 mm). CT scan machines and procedures did not change during study period. The chest CT scans were reviewed blindly by 2 radiologists (U.S. and K.C., with 5 and 15 years of experience in thoracic imaging, respectively) to calculate the lung fibroproliferation CT scan score. The method presented by Kazerooni et al. [24] was used for calculating the CT scan score of lung fibrosis validated with a histological examination. Each lung lobe was scored on a scale of 0–5 for both alveolar and interstitial abnormalities (Additional file 1). In cases of lobectomy or when lung consolidation was observed in more than 75% of a lobe, the score was not used for this lobe. The score was determined on the basis of the area free of consolidation. The crude CT scan score was calculated as the sum of the points of each scored lobe. A non-evaluated lobe was counted as 0 points in the crude score, which could range from 0 to 25 for ground glass and from 0 to 25 for honeycombing. The total crude score ranged from 0 to 50. When a lobe could not be evaluated (important condensation, lobectomy), the CT scan score was corrected for the number of evaluated lobes. The corrected fibroproliferation CT scan score was calculated as the ratio of the sum of the points of each scored lobe to the number of evaluated lobes (corrected score = crude score/number of evaluated lobes). An analysis of 15 CT scans was performed jointly by the two radiologists to calibrate the technique of interpretation and calculation of the score. The agreement of interpretation between the 2 radiologists was analysed by calculating the intraclass correlation coefficient. A collegial analysis between the 2 radiologists occurred in cases of a difference exceeding 10% between the 2 scores calculated by each radiologist. The mean of the scores calculated by the 2 radiologists was used for the statistical analysis. Among all selected patients, 10 patients had open lung biopsy [25] carried out within 7 days after BAL or CT scan.

### Statistical analysis

Descriptive statistics included percentages for categorical variables and medians [interquartile range] for continuous variables according to the distribution. Comparisons between the 2 groups according to lung fibroproliferation for continuous variables were made using a linear mixed model. ICU mortality according to lung fibroproliferation was compared by generalized linear model (generalized equation estimation). Comparisons between the 2 groups for categorical variables were made using Pearson’s Chi-square test or Fisher’s exact test. Correlations between alveolar levels of NT-PCP-III and lung fibroproliferation CT scan scores were carried out by the Spearman test. Using the alveolar levels of NT-PCP-III as a reference (threshold 9 µG/L) [15], we calculated the sensitivity, specificity and predictive values of the CT scan fibrosis scores. The best cut-off value was chosen using Youden’s index. All two-tailed *p* values < 0.05 were considered to be statistically significant. The analyses were performed using SPSS version 20.0 (NY, USA).

## Results

**Fig. 1:**
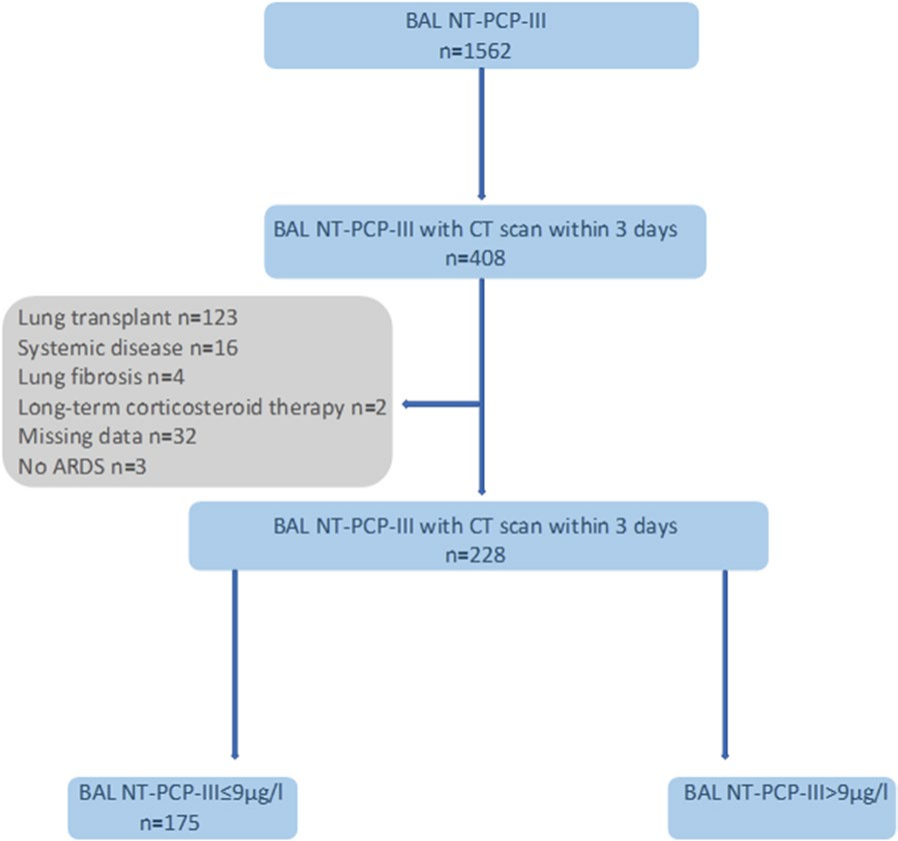
Flow chart. NT-PCP-III: N-terminal peptide for type III procollagen, *ARDS* acute respiratory distress syndrome

One hundred ninety-two patients were included. The flow chart of the study is presented in Fig. 1. Characteristics of the patients are summarized in Table 1. Twenty-eight (14.5%) patients had 2 NT-PCP-III samples associated with 2 different CT scans. Four (2%) patients had 3 NT-PCP-III samples associated with 3 different CT scans. Finally, 228 ‘couples’ (cases) of alveolar NT-PCP-III level and CT scan (within 3 days) were analysed. At the threshold of NT-PCP-III > 9 µG/L, 53 cases (23.2%) had lung fibroproliferation.

**Table 1:** Characteristics of the patients at ARDS onset

Variables	All patients (*n* = 192)
Age (years)	60 [48–69]
Sex [*n* (%) men]	137 (71.4)
Body mass index (kg/m^2^)	25.8 [21.9–29.2]
SAPS II	47 [38–69]
SOFA	8 [7–11]
Cause of ARDS [*n* (%)]	
Pneumonia	104 (54.2)
Aspiration	28 (14.6)
Extra-pulmonary sepsis	28 (14.6)
Others^a^	32 (16.6)
Minute ventilation (L/min)	9.3 [6.6–10.8]
Tidal volume (mL/kg PBW)	6.1 [5.0–6.7]
Respiratory rate (cycle/min)	24 [18–27]
Plateau pressure (cmH_2_O)	25 [21–28]
Driving pressure (cmH_2_O)	13 [9–16]
Total PEEP (cmH_2_O)	10 [8–13]
Respiratory system compliance (mL/cmH_2_O)	26.5 [21.3–36.6]
pH	7.36 [7.29–7.43]
PaO_2_/FiO_2_ (mmHg)	129 [92–197]
PaCO_2_ (mmHg)	43 [36–50]
Ventilator-free days at day 60 (days)	22 [0–46]
Length of ICU stay (days)	24 [12–38]
ICU mortality [*n* (%)]	69 (35.7)

Values are expressed as median [IQR], except for sex, cause of ARDS and ICU mortality [*n* (%)]

*ARDS* acute respiratory distress syndrome, *SAPS II* simplified acute physiologic score, *SOFA* sequential organ failure assessment, *PBW* predicted body weight, *PEEP* positive end-expiratory pressure, *ICU* intensive care unit

^a^Chest trauma with lung contusion (*n* = 18), transfusion-related acute lung injury (*n* = 10), hemoptysis (*n* = 4)

The median delay between the onset of ARDS and the CT scan was 5 [0–14] days, between the onset of ARDS and the BAL was 5 [0–14] days, and between the BAL and the CT scan was 0 [− 1 to 1] days. In 73 (32%) cases, the BAL was performed on the same day as the CT scan; in 71 (31.3%) cases, before; and in 84 (36.9%) cases, after. Patients were comparable in regard to ventilatory parameters and SOFA scores on the days of determination of the NT-PCP-III level and the CT scan (Additional file 2).

**Fig. 2:**
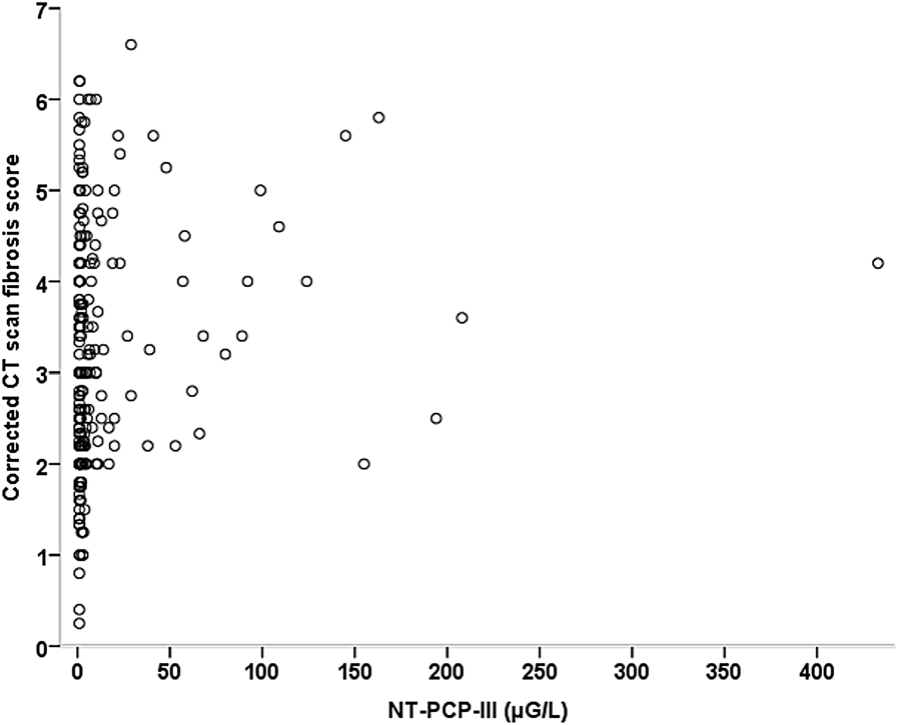
Relationship between corrected CT scan fibrosis score and alveolar levels of NT-PCP-III. *NT-PCP-III* N-terminal peptide for type III procollagen

Values of lung fibroproliferation CT scan scores according to alveolar NT-PCP-III levels are given in Table 2. The median values of the crude and corrected scores were 12 [9–18] and 3.0 [2.2–4.2], respectively. Crude and corrected fibrosis scores were significantly higher in patients presenting alveolar NT-PCP-III > 9 µG/L. CT scan fibrosis scores were significantly but weakly correlated with alveolar NT-PCP-III levels (crude score: Spearman’s *ρ* = 0.178, *p* = 0.007; corrected score: Spearman’s *ρ* = 0.184, *p* = 0.005) (Fig. 2 and Additional file 3).

**Table 2:** CT scan fibrosis scores according to lung fibroproliferation (NT-PCP-III > 9µG/L)

	No lung fibroproliferation (*n* = 175) (NT-PCP-III ≤ 9 µG/L)	Lung fibroproliferation (*n* = 53) (NT-PCP-III > 9 µG/L)	*p* value
Crude fibrosis score	12 [9–17]	14 [11–21]	0.002
Ground glass	7 [5–12]	9 [6–14]	0.007
Honeycombing	4 [4–5]	5 [3–5]	0.015
Corrected fibrosis score	2.8 [2.2–4.0]	3.4 [2.5–4.7]	< 0.001
Ground glass	1.8 [1.2–3.0]	2.3 [1.5–3.3]	0.003
Honeycombing	1 [1–1]	1 [1–1]	< 0.001

Values are expressed as median [IQR]. NT-PCP-III: N-terminal peptide for type III procollagen. Each lobe of the lung was scored on a scale of 0–5 points for both ground glass and honeycombing abnormality. In the case of lobectomy or when consolidation was observed in more than 75% of a lobe, the score was not used for this lobe. The CT scan crude fibrosis score was calculated as the sum of points obtained for each lobe. We corrected the score by reporting it to the number of lobes evaluated (corrected fibrosis score = crude fibrosis score/number of lobes evaluated)

*p* values were calculated by linear mixed model

**Fig. 3:**
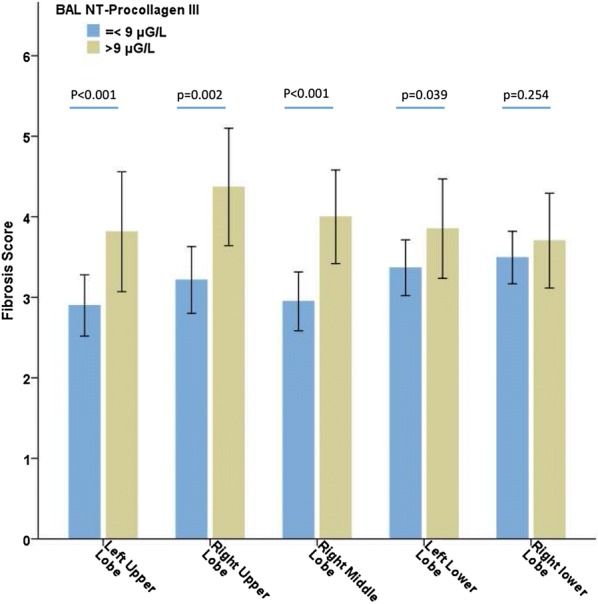
CT scan fibrosis score according to lung segmentation. Error bars represent standard error of mean (SEM), *BAL* bronchoalveolar lavage, *NT-PCP-III* N-terminal peptide for type III procollagen

Figure 3 shows the CT scan fibrosis scores according to lung segmentation and alveolar NT-PCP-III. In 28 (12.3%) cases, the right upper lobe could not be evaluated, as well as 10 (4.4%) cases for the upper left lobe, 27 (11.8%) for the middle lobe, 64 (28.1%) for the lower right lobe, and 54 (23.7%) for the lower left lobe. The intraclass correlation coefficient calculated based on the two radiologists’ results was 0.97 [95% confidence interval (CI) 0.96–0.98; *p* < 0.001]. No relationship was identified between the extent of lung fibroproliferation (determined by alveolar NT-PCP-III or CT scan) and the delay between ARDS onset and of this assessment.

When the alveolar level of NT-PCP-III was used to define lung fibroproliferation with a threshold of > 9 µG/L, we showed that the corrected CT scan fibrosis score had a sensitivity of 68% (95% CI 54–80), a specificity of 50% (95% CI 43–58), a positive predictive value of 29% (95% CI 22–38), and a negative predictive value of 71% (95% CI 62–78). The crude CT scan fibrosis score had a sensitivity of 47% (95% CI 34–61), a specificity of 66% (95% CI 58–73), a positive predictive value of 29% (95% CI 20–40) and a negative predictive value of 71% (95% CI 60–80).

ARDS characteristics, ventilatory parameters and outcome according to alveolar levels of NT-PCP-III (threshold > 9 µG/L) and corrected fibrosis scores (threshold ≥ 3.0) are presented in Table 3. Eleven (5.7%) patients received corticosteroids for lung fibroproliferation treatment according to the protocol described by Meduri et al. (methylprednisolone 2 mg/kg) [10]. No patient was treated prior to the BAL or CT scan.

**Table 3:** Characteristics of ARDS, ventilatory parameters and outcome according to lung fibroproliferation and CT scan fibrosis score

	CT scan day			BAL day		
	Corrected score < 3 (*n* = 105)	Corrected score ≥ 3 (*n* = 123)	*p*	NT-PCP-III ≤ 9 µG/L (*n* = 175)	NT-PCP-III > 9 µG/L (*n* = 53)	*p*
Minute ventilation (L/min)	10.2 [7.9–11.8]	10.3 [8.8–11.7]	0.66	9.6 [8.1–11.0]	9.9 [8.37–12.0]	0.89
Tidal volume (mL/kg PBW)	6.3 [5.5–7.3]	6.3 [4.9–7.3]	0.68	6.3 [5.7–6.9]	6.1 [5.3–6.8]	0.82
Respiratory rate (cycle/min)	24 [20–28]	25 [22–30]	0.39	24 [20–28]	25 [22–30]	0.34
Plateau pressure (cmH_2_O)	23 [20–26]	26 [22–28]	0.08	25 [22–27]	26 [20–29]	0.91
Driving pressure (cmH_2_O)	12 [10–15]	14 [12–17]	0.77	14 [11–16]	14 [12–18]	0.69
PEEP (cmH_2_O)	10 [7–12]	10 [8–12]	0.76	10 [8–12]	10 [8–14]	0.24
Respiratory system compliance (mL/cmH_2_O)	33 [24–40]	24 [18–34]	0.10	28 [22–37]	26 [19–35]	0.84
pH	7.39 [7.32–7.43]	7.38 [7.32–7.44]	0.90	7.39 [7.32–7.44]	7.37 [7.31–7.44]	0.89
PaO_2_/FiO_2_ (mmHg)	162 [111–224]	166 [119–213]	0.75	160 [120–221]	143 [111–187]	0.19
PaCO_2_ (mmHg)	44 [37–50]	44 [35–50]	0.50	43 [36–40]	46 [40–56]	0.07
SOFA	7 [5–9]	7 [5–9]	0.25	7 [5–9]	7 [6–9]	0.57
Ventilator-free days at day 60 (days)	31 [0–52]	0 [0–38]	0.001	23 [0–43]	0 [0–45]	0.25
Length of ICU stay (days)	23 [12–41]	28 [15–43]	0.21	27 [14–38]	30 [14–55]	0.31
ICU mortality [*n* (%)]	29 (27.6)	54 (43.9)	0.02	55 (31.4)	28 (52.8)	0.01

Values are expressed as median [IQR]

*ARDS* acute respiratory distress syndrome, *BAL* bronchoalveolar lavage, *PBW* predicted body weight, *PEEP* positive end-expiratory pressure, *SOFA* sequential organ failure assessment, *NT-PCP-III* NT-peptide for type III procollagen

*p* values were calculated by linear mixed model, excepted for ICU mortality by generalized linear model (generalized equation estimation)

Ten patients (among the 228 selected patients) had open lung biopsy for histological assessment. Table 4 reports the alveolar levels of NT-PCP-III and the CT scan fibrosis scores according to the histological lung fibroproliferation. The number of lobes evaluated by CT scan was same in the group with fibroproliferation or without histological fibroproliferation (4 [2–4] and 4 [4–4], *p* = 0.96, respectively). The median delay between the onset of ARDS and OLB was of 7 [5–23] days, between LBA and OLB 3 [2–4] days, and between CT scan and OLB 3 [2–5] days.

**Table 4:** Relationship between the level of alveolar NT-PCP-III, CT scan fibrosis score and the histological lung fibroproliferation on open lung biopsies

	No histological fibroproliferation (*n* = 7)	Histological fibroproliferation (*n* = 3)	*p* value
NT-PCP-III (µG/L)	1 [1–1]	13 [10–99]	0.01
Corrected fibrosis score	2.5 [1.0–3.0]	3.0 [2.8–5.0]	0.14
Ground glass score	1.5 [0.3–2.0]	2.0 [1.8–4.0]	0.14
Honeycombing score	1.0 [1.0–1.0]	1.0 [1.0–1.0]	0.52

Ten patients have an open lung biopsy within 7 days around the BAL and the chest CT scan. Values are expressed as median [IQR]

*NT-PCP-III* N-terminal peptide for type III procollagen, *BAL* bronchoalveolar lavage

## Discussion

Alveolar NT-PCP-III and CT scan are useful diagnostic tools for lung fibroproliferation during ARDS. When the alveolar level of NT-PCP-III is used as a surrogate marker of histological lung fibroproliferation, CT scan fibrosis scores are significantly higher in patients showing active pulmonary fibroproliferation. Nevertheless, the sensitivity, specificity and predictive values of the CT scan fibrosis scores were poor (all ≤ 71%) compared to the alveolar levels of NT-PCP-III. Pulmonary condensation is the main limitation to diagnosing fibroproliferation by chest CT scan during ARDS.

This study is the first to describe the radiologic damage to the lungs caused by fibroproliferation during ARDS according to the alveolar levels of NT-PCP-III [15, 26]. Our results were drawn from a large series; however, these should be considered as preliminary and need to be interpreted cautiously. Indeed, the significant but weak correlation between the CT scan fibrosis scores and alveolar NT-PCP-III levels could be explained by the fact that the BAL was performed in the most condensed lobe of the thoracic X-ray (most of the time, one of the lower pulmonary lobes), whereas the lower lobes could not be evaluated by CT scan because of the condensations. We showed that the CT scan fibrosis scores in the right lower lobes were not significantly different according to alveolar NT-PCP-III levels (Fig. 3). Pulmonary condensation was the main limitation for the assessment of the CT scan fibrosis score.

The second hypothesis to explain the low correlation between fibrosis scores and NT-PCP-III levels may be related to the chronological change in the lung fibroproliferation process. Alveolar NT-PCP-III is an early marker of lung fibroproliferation. As a collagen III precursor, alveolar NT-PCP-III precedes the deposition of type 1 collagen associated with histological lung fibrosis [26–28]. CT scans describe lung fibrosis signs that correspond to alterations in the pulmonary structure. We showed that when CT scan fibrosis scores are high, plateau pressures and respiratory system compliance tend to be higher and lower, respectively (Table 3). These results agree with the study by Burnham et al. [23], which showed that plateau pressures and static respiratory system compliance were correlated with the presence of crosslinking, ground glass and bronchiectasis on CT scans on the 14th day of ARDS. This difference in the age assessment of the lung fibroproliferative process could explain the weak correlation found in our study. Indeed, in the present study, alveolar levels of NT-PCP-III were measured relatively early in the ARDS history (5 [0–14] days). However, Forel et al. [15] showed that high alveolar levels of NT-PCP-III on the 6th day (median) of ARDS onset are associated with histological lung fibroproliferation on lung biopsy performed on the 10th day (median). The different stage in the fibroproliferative process could explain why we did not find a significant difference in ventilatory parameters based on alveolar NT-PCP-III, including respiratory system compliance. In fact, it is the deposition of collagen I within the lung tissue that causes a decrease in elasticity and a reduction in lung compliance. Demoule et al. [29] showed an inversely proportional logarithmic relationship between respiratory system compliance and alveolar NT-PCP-III. We not observed a similar relationship. This difference could be explained by the use of different assay techniques to determine NT-PCP-III in Demoule and our study. The reduction in respiratory system compliance remains a complex matter of interpretation. Pulmonary volume reduction during ARDS appeared to be the main mechanism for this reduction [29, 30], but lung damage and thoracic wall involvement also played a role, which was not analysed in our study. Moreover, in the present study, we registered respiratory system compliance on the day of BAL and CT scan, but we not analysed its changes during time which are relevant in the clinical practice.

Our study has others limitations. The CT scan fibrosis score used was initially described for idiopathic pulmonary fibrosis [24]. Pulmonary condensation, which is common in ARDS patients, was the main limitation of the CT scan fibrosis evaluation in our study. Concerning alveolar abnormalities, ground glass is a non-specific sign due to ARDS and to any cause of partial filling of the alveolar air space [31]. Concerning interstitial abnormalities, only a few patients had honeycombing. This low proportion is in line with the results of Ichikado et al. [22] (in fact, none of their patients had honeycombing), but the difference could be explained by the early time period of the CT scan in their study (24 h) compared to ours (5 [0–14] days). When honeycombing was observed, it was not possible to identify whether distinct abnormalities existed prior to or were due to ARDS when no previous chest CT examination was found. Septal thickening is a non-specific sign and can be the result of numerous causes: lung fibrosis due to ARDS, or any venous, lymphatic or infiltrative diseases. Despite the rigorous selection of patients in our study, this bias was possible. Others CT scan fibrosis scores are described in the literature [22, 23], but, unlike the score used [24], none were validated by histological data. To date, the alveolar level of NT-PCP-III can be considered as the best surrogate marker to an OLB for the diagnosis of lung fibroproliferation [15]. However, the treatment by corticosteroids of lung fibroproliferation remains discussed [10, 13]. Steinberg et al. showed a trend in mortality reduction in the ARDS patients with high alveolar levels of NT-PCP-III and treated by methylprednisolone. We started a confirmation study (PROCOCO, NCT03371498).

## Conclusions

When the alveolar level of NT-PCP-III was used as a surrogate of histological lung fibroproliferation, the lung CT scan fibrosis score was significantly higher in patients with active lung fibroproliferation. Nevertheless, pulmonary condensation is the main limitation to diagnosing fibroproliferation by CT scan during ARDS. Further studies are needed to determine the position of the CT scan in the strategy of early diagnosis for lung fibroproliferation in ARDS.

## Additional files

**Additional file 1.** CT scan scoring system for lung fibrosis.

**Additional file 2.** Comparison of ventilatory parameters and organ dysfunction score on CT scan and BAL days.

**Additional file 3.** Relationship between crude CT scan fibrosis score and alveolar levels of NT-PCP-III.

## Abbreviations

ARDS: acute respiratory distress syndrome
BAL: bronchoalveolar lavage
CT scan: computerized tomography scan
NT-PCP-III: N-terminal-peptide type III procollagen
OLB: open lung biopsy
SOFA: sequential organ failure assessment
