# Supplementary material for: Chest CT scan and alveolar procollagen III to predict lung fibroproliferation in acute respiratory distress syndrome
Source: Ann Intensive Care. 2019 Mar 27;9:42. doi: 10.1186/s13613-019-0516-9 (PMC6437222; doi:10.1186/s13613-019-0516-9)
Supplement: Supplementary file 1 — Additional file 1. CT scan scoring system for lung fibrosis. [file 13613_2019_516_MOESM1_ESM.docx]

**Additional file 1. CT scan scoring system for lung fibrosis**

| Score | Features |
| --- | --- |
| Ground glass score | |
| 0  1  2  3  4  5 | No ground glass opacity  Ground-glass opacity involving ≤5% of the lobe  Ground-glass opacity involving 5-<25% of the lobe  Ground-glass opacity involving 25-49% of the lobe  Ground-glass opacity involving 50-75% of the lobe  Ground-glass opacity involving >75% of the lobe |
| Honeycombing score | |
| 0  1 2 3 4 5 | No fibrosis  Interlobular septal thickening: no discrete honeycombing  Honeycombing(with or without septal thickening) involving <25% of the lobe  Honeycombing(with or without septal thickening) involving 25-49% of the lobe  Honeycombing(with or without septal thickening) involving 50-75% of the lobe  Honeycombing(with or without septal thickening) involving >75% of the lobe |

*Each lobe of the lung was scored on a scale of 0 to 5 points for both ground glass and honeycombing abnormality. In case of lobectomy or when consolidation was observed in more than 75% of a lobe, the score was not used for this lobe. The CT scan crude fibrosis score was calculated as the sum of points obtained for each lobe. We corrected the score by reporting it to the number of lobes evaluated (Corrected fibrosis score = Crude fibrosis score / number of lobes evaluated).*

*From* *Kazerooni EA, Martinez FJ, Flint A, Jamadar DA, Gross BH, Spizarny DL, et al. Thin-section CT obtained at 10-mm increments versus limited three-level thin-section CT for idiopathic pulmonary fibrosis: correlation with pathologic scoring. Am J Roentgenol. oct 1997;169(4):977‑83.*
