# Supplementary material for: Chest CT scan and alveolar procollagen III to predict lung fibroproliferation in acute respiratory distress syndrome
Source: Ann Intensive Care. 2019 Mar 27;9:42. doi: 10.1186/s13613-019-0516-9 (PMC6437222; doi:10.1186/s13613-019-0516-9)
Supplement: Supplementary file 2 — Additional file 2. Comparison of ventilatory parameters and organ dysfunction score on CT scan and BAL days. [file 13613_2019_516_MOESM2_ESM.docx]

**Additional file 2. Comparison of ventilatory parameters and organ dysfunction score on CT scan and BAL days**

|  | CT scan day  (n=228) | BAL day  (n=228) | *p value* |
| --- | --- | --- | --- |
| Minute ventilation, L/min | 10.2 [8.3-11.8] | 9.7 [8.1-12.0] | 0.14 |
| Tidal volume, mL/kg PBW | 6.3 [5.2-7.3] | 6.2 [5.5-6.8] | 0.61 |
| Respiratory rate, cycle/min | 25 [20-30] | 25 [20-29] | 0.44 |
| Plateau pressure, cmH_2_O | 25 [21-28] | 25 [21-28] | 0.24 |
| Driving pressure, cmH_2_O | 13 [10-16] | 14 [11-16] | 0.19 |
| Total PEEP, cmH_2_O | 10 [8-12] | 10 [8-12] | 0.76 |
| Respiratory system compliance, mL/cmH_2_O | 29 [21-39] | 27 [22-37] | 0.57 |
| pH | 7.38 [7.32-7.44] | 7.38 [7.32-7.44] | 0.83 |
| PaO_2_/FiO_2_ mmHg | 164 [116-218] | 157 [120-213] | 0.11 |
| PaCO_2_, mmHg | 44 [36-50] | 44 [37-52] | 0.05 |
| SOFA | 7 [5-9] | 7 [5-9] | 0.28 |

*Values are expressed as Median [IQR]. BAL: bronchoalveolar lavage, PBW: predicted body weight, PEEP: positive end-expiratory pressure, SOFA: sequential organ failure assessment*
