# Supplementary material for: Chest CT scan and alveolar procollagen III to predict lung fibroproliferation in acute respiratory distress syndrome
Source: Ann Intensive Care. 2019 Mar 27;9:42. doi: 10.1186/s13613-019-0516-9 (PMC6437222; doi:10.1186/s13613-019-0516-9)
Supplement: Supplementary file 3 — Additional file 3. Relationship between crude CT scan fibrosis score and alveolar levels of NT-PCP-III. [file 13613_2019_516_MOESM3_ESM.docx]

**Additional file 3. Relationship between crude CT scan fibrosis score and alveolar levels of NT-PCP-III**


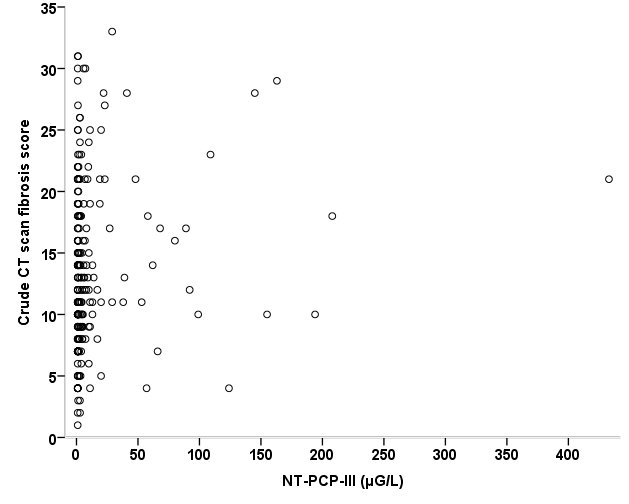


*NT-PCP-III: NT-peptide for type III procollagen*
